# Application of D-Crustacean Hyperglycemic Hormone Induces Peptidases Transcription and Suppresses Glycolysis-Related Transcripts in the Hepatopancreas of the Crayfish *Pontastacus leptodactylus* — Results of a Transcriptomic Study

**DOI:** 10.1371/journal.pone.0065176

**Published:** 2013-06-19

**Authors:** Chiara Manfrin, Moshe Tom, Gianluca De Moro, Marco Gerdol, Corrado Guarnaccia, Alessandro Mosco, Alberto Pallavicini, Piero Giulio Giulianini

**Affiliations:** 1 Dipartimento di Scienze della Vita, Università di Trieste, Trieste, Italy; 2 Israel Oceanographic and Limnological Research, Haifa, Israel; 3 ICGEB, International Centre for Genetic Engineering and Biotechnology, AREA Science Park, Trieste, Italy; International Centre for Genetic Engineering and Biotechnology, Italy

## Abstract

The crustacean Hyperglycemic Hormone (cHH) is a neuropeptide present in many decapods. Two different chiral isomers are simultaneously present in Astacid crayfish and their specific biological functions are still poorly understood. The present study is aimed at better understanding the potentially different effect of each of the isomers on the hepatopancreatic gene expression profile in the crayfish *Pontastacus leptodactylus*, in the context of short term hyperglycemia. Hence, two different chemically synthesized cHH enantiomers, containing either L- or D-Phe^3^, were injected to the circulation of intermolt females following removal of their X organ-Sinus gland complex. The effects triggered by the injection of the two alternate isomers were detected after one hour through measurement of circulating glucose levels. Triggered changes of the transcriptome expression profile in the hepatopancreas were analyzed by RNA-seq. A whole transcriptome shotgun sequence assembly provided the assumedly complete transcriptome of *P. leptodactylus* hepatopancreas, followed by RNA-seq analysis of changes in the expression level of many genes caused by the application of each of the hormone isomers. Circulating glucose levels were much higher in response to the D-isoform than to the L-isoform injection, one hour from injection. Similarly, the RNA-seq analysis confirmed a stronger effect on gene expression following the administration of D-cHH, while just limited alterations were caused by the L-isomer. These findings demonstrated a more prominent short term effect of the D-cHH on the transcription profile and shed light on the effect of the D-isomer on specific functional gene groups. Another contribution of the study is the construction of a *de novo* assembly of the hepatopancreas transcriptome, consisting of 39,935 contigs, that dramatically increases the molecular information available for this species and for crustaceans in general, providing an efficient tool for studying gene expression patterns in this organ.

## Introduction

The freshwater astacid *Pontastacus leptodactylus*, commonly called narrow-clawed crayfish, inhabits Western Asian and Eastern European lakes and watercourses. Its reproductive season includes mating from December to January and spawning in January. Eggs are incubated glued to the female pleopods till July [1]. Ovarian development takes place from June to November [2]. Induced molt through chirurgical intervention in *P. leptodactylus* maintained at 19°C, led to a premolt period of 17 days ending with ecdysis (personal observations of the present authors). Adults naturally shed their exoskeleton in summer just after hatching, even though some of them molt also in autumn [3].

Crustacean hyperglycemic hormones (cHHs) are a pleiotropic crustacean-specific neuropeptide family, functioning in a variety of physiological processes, recently reviewed by several authors [4], [5], [6], [7], [8]. The cHH family is divided into two subfamilies on the basis of their primary structure: (a) the cHH subfamily and (b) the molt-inhibiting hormone (MIH), the mandibular organ inhibiting hormone (MOIH) and the vitellogenesis/gonad-inhibiting hormone (V/GIH) subfamily. The translated neuropeptides in the tissues as well as their isoforms derived from post-translational modifications and their modes of action have only been partially described. Hence, a neurohormone name does not necessarily imply its entire range of functions. Several cHH variants occasionally co-exist in a single species. The variability can emerge either from a different primary sequence or from different post-translational modifications [9], [10]. Recently, chirality was observed also in a lobster VIH due to L to D alteration in the fourth N terminal amino acid, a tryptophan residue [11] demonstrating larger extent of the phenomenon in crustaceans. cHHs are produced in the neurosecretory perikarya sited in the medulla terminalis of the optic ganglion, located in the crustacean eyestalk and named X-organ. The X-organ secretes the neuropeptides into the hemal sinus gland and the entire neuroendocrine complex is abbreviated XOSG. Structurally, the cHH prepropeptide is composed of a signal peptide, a cHH precursor related peptide (CPRP) and a mature peptide of 72 amino acids. The role of the CPRP is still unknown, but CPRP structures, post-translational modifications and individual-related distribution have already been described [12], [13]. The mature cHH contains six cysteine residues that form three disulfide bridges and potentially possesses an amidated C-terminus and a pyroglutamate blocked N-terminus [6]. Documented physiological processes influenced by the eyestalk ablation are vitellogenesis [14], food intake, digestion, and nutrient transport [15], molting [16], metabolism of lipids [17], [18], regulation of glucose and proteins in hemolymph [17], [19], hydromineral balance, regeneration and pigment regulation [20]. Several cHH family members generally have an inhibitory effect, as the removal of the XOSG causes induction of both molt and reproduction. cHHs are produced also in other tissues: the pericardial organ, the subesophageal ganglia, and the fore- and hindguts. The more comprehensively studied action of the cHH is the regulation of carbohydrate metabolism. Its secretion follows a circadian rhythm, with a low concentration during the day which increases in the first hours of the night, and it is correlated to a similar daily pattern of the glycemia [21]. The injection of cHH induces a fast pharmacological hyperglycemic response in treated animals. Apart from glucose metabolism, cHH mediates other metabolic functions of the hepatopancreas, the site of synthesis and secretion of digestive enzymes (amylases, proteases, lipases and others) [22], [23], [24]. It is also involved in metabolism of proteins, lipids, and carbohydrates [25], as well as in the catabolism of organic compounds and in detoxification [26], [27]. Indeed, cHH stimulates amylase secretion [28], and the release of free fatty acids and phospholipids [18] from the midgut gland. D-cHH is also involved in the control of molt, exerting its function by inhibiting the synthesis of ecdysone in the Y-organ and having an activity 10 times higher than L-cHH [29]. Contrasting activities were reported for the CHH regulation of reproduction, probably due to species specificity [30], [31], [32], [33].

The structure of the cHH genes, the derived precursors and peptides have been recently reviewed by Webster and colleagues [7], while the dynamics of biosynthesis and release of cHH isoformes in *Orconectes limosus* have been clarified by Ollivaux and Soyez [34]. cHH peptide sequences were *de novo* sequenced through a multifaceted mass spectrometry approach by Jia and colleagues [35].

Although *P. leptodactylus* is not considered a model organism, it is one of the most studied species among decapods with about 143 published papers regarding its physiology [1], [36], [37], [38], its resistance to different types of pollution and stress conditions [39], [40], [41] and concerning the cHH and its variety of functions [42], [43], [44], [45]. Unfortunately this variety of studies does not reflect the fast progress of genomics and transcriptomics. Back in 1988 Sedlmeier [28] demonstrated that cHH increased amylase secretion from the hepatopancreas of *O. limosus*. He also identified the involvement of the second messengers: cAMP, cGMP and calcium ion and a participating adenylate cyclase. However, no effect on gene expression was examined and no distinction was made between the two *O. limosus* cHH isomers, discovered later [34]. The present study is aimed at defining the effect of cHH at the hepatopancreas transcriptome level distinguishing the putatively different effect of the two stereomers.The effect of the two isomers of cHH on gene expression in relation to their variety of assigned functions in the relevant tissues is still lacking. The working hypothesis for this study assumed two alternate or complementary effects of each of the two hormones: changes in gene expression or activation/inactivation of participating proteins. Elevation of circulating glucose level upon application of D and L-cHH to eyestalkless *P.leptodactylus* even one hour post-injection is well documented [42]. Hence, the study is aimed at examining the two alternatives during this short time span by simultaneously measuring the circulating glucose levels and changes of gene expression in the hepatopancreas applying a transcriptome approach. Beside this gene expression experiment, the construction of a *de novo* assembly of the hepatopancreas transcriptome, dramatically increased the molecular information available for this species and undoubtedly provides a useful tool for further gene expression experiments in this organ.

## Materials and Methods

### Crayfish maintenance and experimental design

Adult *P. leptodactylus* were bought from a local dealer on July, 2011. They were kept for two weeks before the experiment in 120 L tanks provided with closed circuit filtered and thoroughly aerated tap water at ∼18°C and were fed by fish pellets (Sera granular, Heisenberg, Germany) three times per week. Only females with no abdominally incubated ova were taken for the induction experiment. The females were at the end of the ova incubation period.

The chemical synthesis of the peptides and the glucose level induction protocol were accomplished according to Mosco et al. [42] with a few modifications detailed below. The two synthetic cHH hormone isomers, D-cHH and L-cHH, were injected to the circulation. Thirty-two *P. leptodactylus* females were divided into four groups each composed of 8 females. Two of the groups were injected with D- and L-cHH, respectively (0.5 µg/female in 100 µl PBS). A control group was sham-injected by the hormone carrier (S). Bilateral ablation of the XOSG, aimed at prevention of any possible interference due to endogenous cHH was performed 48 hours before the injections, and a fourth group of naïve females (N) was added to the experiment as control just before injections. All 32 females were sacrificed one hour post-injection. The incubation period included ten minutes of anesthesia in ice before sacrifice and all efforts were made to minimize the suffering of the animal. Immediately prior to the hormonal injection the females were bled for the evaluation of the pre-induction hemolymphatic glucose level. A second bleeding was performed just before sacrifice. Hemocytes were pelleted from the sampled hemolymph and the serum was kept on ice for later glucose measurement, which was performed using a glucose oxidase method (glucose liquid mono reagent, Hospitex diagnostics, Italy). The statistical analyses of glucose levels recorded in the four groups and females' morphometric parameters were performed using R software, version 2.14.1 [46] as follows: the normality of data was checked with a Shapiro-Wilk test and homogeneity of variance across groups was checked with a Bartlett test. The null hypotheses of both tests could not be rejected. Hence, differences of glucose levels among the experimental groups were tested using non-parametric statistics, Kruskal-Wallis rank sum test with post-hoc Wilcoxon rank sum test pairwise comparisons with Bonferroni correction. Box and whiskers plots were drawn with the boxplot command of R. Glucose levels are expressed as mean ± standard error. Carapace length was measured from its posterior edge to the base of the eye cavity just before sacrifice and a sample of hepatopancreatic tissue of ∼5×5×5 mm was dissected out and immediately snap-frozen in liquid nitrogen. The gastroliths were also taken for molt stage evaluation according to Shechter et al. [47] using the molt mineralization index (MMI) (gastrolith width/crayfish carapace length).

### Ethical Note

The experiments comply with the current laws of Italy, the country in which they were done. No specific permits were required for the studies that did not involve endangered or protected species. Individuals were maintained in appropriate laboratory conditions to guarantee their welfare and responsiveness. After the experiments were completed, crayfish were sacrificed by hypothermia.

### RNA extraction and sequencing

Twelve out of 32 females were randomly chosen for the RNA sequencing. RNA was extracted from frozen tissues, homogenized in TriReagent RNA isolation solution (Sigma-Aldrich, Cat UN2821) following the manufacturer's instructions. The resulted RNAs were further purified using the RNeasy kit (Qiagen, manufacturer's instructions). The RNA level was quantified by spectrophotometer and its quality was examined using capillary electrophoresis (BioAnalyzer 2100, Agilent).

RNA sequencing was carried out at the Applied Genomics Institute (IGA, Udine, Italy), on an Illumina sequencer HiSeq2000. The hepatopancreas reference transcriptome assembly was derived from a 2×100 bp paired-end sequencing performed on a cDNA library obtained from equimolar amounts of RNA taken from all experimental females. Gene expression was evaluated using the single-end 50 bp Illumina sequencing from the 12 distinct RNAs.

### Sequences analysis

The processing and analysis of the obtained raw sequences was carried out using the CLC Genomics Workbench 4.5 software (CLC Bio, Aarhus, Denmark). Raw sequence reads were trimmed according to base calling quality. The resulting 2×100 bp sequence reads were assembled assuming a paired reads distance between 100 and 600 base pairs, and setting the penalties for mismatches to 2, insertions and deletions to 3 and similarity and length fraction to 0.9 and 0.5, respectively. The minimum allowed assembled contig length was set to 200 bp. The obtained contig assembly served as a comprehensive reference for the functional genomics RNA-seq analysis [48] of the *P. leptodactylus* hepatopancreas. We determined the presumptive amount of conceptual full length transcripts by using the Full-lengther Next webtool [49] considering alignments starting before the 10^th^ aa and with an e-value below 1e-04. Filtered contigs displaying an average coverage lower than 25× of mapped 50 bp reads were discarded prior to the RNA-seq analysis for creating a robust set of contigs not subject to random expression fluctuations, corresponding to the 95^th^ percentile of the genes expressed in the hepatopancreas throughout the experiment.

The 50 bp sequencing reads from the 12 different samples (namely, N1, N2, N3, S1, S2, S3, L1, L2, L3, D1, D2 and D3) were individually mapped on the filtered reference set using the RNA-seq procedure, considering a maximum number of mismatches cost of 3 and a maximum of 10 hits for a read. The expression values were calculated based on unique gene reads. The Baggerly's test [50] was used to identify statistically significant differential expression using S as a reference group; a FDR corrected p-value<0.01 was set as threshold of significant differential expression [51] and an additional minimum threshold of fold change of 2 was also used. The similarity among the profiles obtained from this study was examined by hierarchical clustering (complete linkage, average linkage and single linkage) using the Pearson correlation coefficient as a distance measure. An alternative clustering was performed by Principal Component Analysis (PCA). The raw Illumina reads were stored at the NCBI Sequence Read Archive (SRA: SRR650486), whereas 39,935 assembled contigs were deposited at NCBI Transcriptome Shotgun Assembly (TSA: GAFY00000000).

### Transcripts annotation and their expression pattern

Differentially expressed transcripts were characterized with the Blast2Go platform [52], [53]. The characterized parameters were: resemblance to genes with known function using BLASTx algorithm [54] against the NCBI non-redundant protein databases applying an e-value cut-off of 10^6^. The default Blast2Go similarity annotations were also checked manually against the list of resemblances obtained for each contig by BLASTx, to conform to the UniProt nomenclature guidelines and to select the annotations in a more educated manner, based on the GO terms and domain characterization described below. Blast2Go was used also to assign Gene Ontology (GO) functional terms [55] and Interpro domains annotations [56] to the contigs, with default settings. The GO and Interpro annotation outputs were modified with scripts developed in-house and imported into the CLC Genomics Workbench 4.5 environment for implementing the hypergeometric test [57] on the annotations. Significantly altered GO terms and Interpro domains were detected with this test considering a p-value threshold of 0.01 and a difference between observed and expected >1.

## Results and Discussion

### Morphological and physiological state of the animals

**Figure 1:**
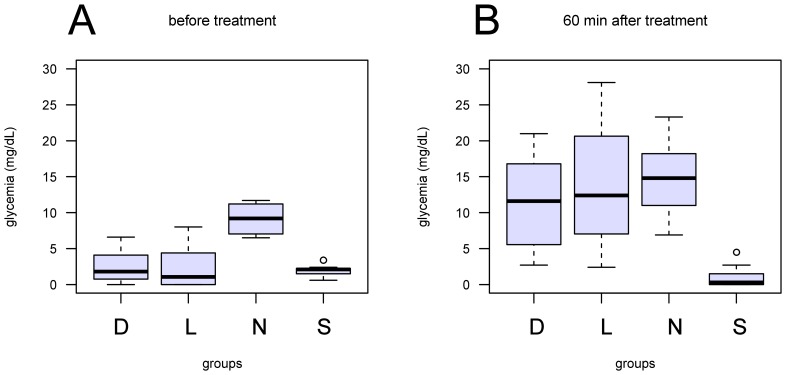
Box plot of the glycemia values obtained in the four groups S – sham injected, N – intact, L – L-cHH-injected, D- D-cHH-injected at the beginning of the experiment and before sacrifice, one hour post-injection.

The morphometric characteristics and the molt stage of the experimental females as well as their hemolymphatic glucose levels before injection and at their sacrifice are presented in **Table 1**. The glucose level of all the 3 groups of eyestalk-less females was highly significantly lower than the native ones (Wilcoxon rank sum test: p<0.01; **Figure 1A**), but after an hour the injection of both cHHs was able to restore glycemia to almost normal levels (D-cHH and L-cHH injected animals vs native animals, Wilcoxon rank sum test: p = 1; **Figure 1B**). On the contrary, the sham-injected animals still presented a significantly lower glycemia compared to all other groups (Wilcoxon rank sum test: p<0.02). The carapace length was similar among the four experimental groups, namely N, S, L and D (Wilcoxon rank sum test: p>0.05). All females were at intermolt with MMI<0.01 and all ovaries were immature according to Hubenova et al. [2].

**Table 1:** Morphometric characteristics of the experimental females, their hemolymphatic glucose levels before injection and at their sacrifice.

Experimental group [n = 8 in each group]	Carapace length [mm]	*MMI – Gastrolith width/Carapace length [mm/mm]	Glucose levels [mg/dL]	
			0 min.	60 min
Native	39.4±1.2	0.0067±0.0048	9.14±0.75	14.78±1.93
Sham-injected	39.4±2.4	0.0094±0.0086	1.94±0.33	1.01±0.59
L-cHH-injected	41.5±2.0	0.0081±0.0065	2.36±1.10	13.84±3.13
D-cHH-injected	39.9±1.7	0.0025±0.0001	2.49±0.83	11.45±2.32

* MMI – molt mineralization index.

### De novo assembly of the* P. leptodactylus* hepatopancreas transcriptome

The Illumina 2×100 bp sequencing of the hepatopancreas of adult *P. leptodactylus* females, generated 296,112,296 nucleotide reads. The number was reduced to 289,731,590 after quality trimming. The *de novo* assembly by the CLC Genomic Workbench produced 42,187 contigs with an average length of 802 bp. This assembly was used as a reference transcript library for subsequent analyses. A total of 9,474 contigs longer than 1 Kb was obtained, while the longest contig almost reached 15 Kb in length. **Table 2** summarizes the trimming and assembly statistics. The raw Illumina reads were stored at the NCBI Sequence Read Archive (SRA: SRR650486), whereas 39,935 assembled contigs were deposited at NCBI Transcriptome Shotgun Assembly (TSA: GAFY00000000). The reduction in number of deposited contigs was obliged by the TSA quality control process which includes additional trimming of primers, shortening of long N rows artificially produced by the definition of the assembly parameters which rendered some contigs shorter than 200 bps. Moreover, some genes suspected to be introduced by contamination were deleted as well. Through the Full-Lengther Next webtool [49] we determined the presumptive percentage of the full length transcripts present in our library. Hence, 1/3^rd^ (32.2%) of the transcripts were putatively assembled to their full length, 1/3^rd^ (32.6%) covered either the N-terminal or the C-terminal region, whereas the remaining 35.2% consisted of internal fragments.

**Table 2:** Trimming statistics and assembly summary of the Reference dataset.

Trimming statistics	
Number of reads before trimming	296,112,296
Number of reads after trimming	289,731,590
Paired-end reads after trimming	283,857,480
Single reads after trimming	5,874,110
Sequences discarded during trimming	6,380,706
Average length before trimming	101.0
Average length after trimming	97.2

Assembly statistics	
Assembly size [bps]	33,838,039
Total number of contigs	42,187
Number of contigs submitted to TSA	39,935
N50	1208
N80	454
N90	318
Mean contig length	802
Median contig length	449
Longest contig	14,918
Number of contigs longer than 1 Kb	9,474
GC content	40.44%

N50, 80 and 90 are the quantiles corresponding to the 50, 80 and 90 percentiles, respectively.

From the output of Blast2Go we checked the thirteen Top-hits species sharing similarity with the *P. leptodactylus* reference library. The two most represented species were *Daphnia pulex* (Crustacea) and *Tribolium castaneum* (Insecta). The first thirteen BLAST top-hits species are reported in **Figure S1**. To date, the only crustacean whose genome has been fully sequenced is the branchiopod *Daphnia pulex* [58]. Even though a few deep transcriptome sequencing approaches were applied to decapods [59], [60], no study has ever targeted Astacidae. An astacoid epithelial assembly from *Cherax quadricarinatus*, performed by 454 was simultaneously prepared by the present co-authors and their collaborators and will be soon released for the public (TSA: GADE00000000). At present, only 275 nucleotide sequences belonging to the genus *Pontastacus* are stored at NCBI, while only 67 originated from *Pontastacus leptodactylus*. Next generation sequencing greatly simplifies large-scale molecular studies of non-model organisms, and this transcriptome sequencing with the assembly of 39,935 contigs, represents the first large-scale sequencing approach in this genus, as well as a remarkable contribution to the genetic knowledge of decapods, paving the way to straightforward comparative molecular studies on non-model crustaceans.

### RNA-seq analysis of cHH isomer effects

An average of 12.7±5.2 million short reads were obtained from the sequencing of RNAs extracted from the hepatopancreas of each sampled female. An average of 50.8±6.9% reads were mapped to the reference transcriptome. **Table 3** reports in detail the number of reads obtained from each adult female. The RNA-seq mappings of the 12 sequenced samples (N1, N2, N3, S1, S2, S3, L1, L2, L3, D1, D2 and D3) were used for the gene expression analysis. Only a selected set of 6,860 contigs with an average coverage >25× was used for the analysis, reducing the noise potentially caused by the high number of fragmented or poorly expressed transcripts, not strictly related to the hepatopancreas, as highlighted by the Full-lengther analysis. This selected set of transcripts accounts for about 96% of the total expression in this organ during the present experiment. The stringency applied to the contigs selection (FDR corrected p-value <0.01 and a weighted fold change at least of 2), identified the prominently differentially expressed transcripts in the pairwise comparisons, by using the S group as reference for all the analyses. **Table 4** summarizes the numbers of differentially expressed genes while the complete list of differentially expressed genes is available in **Table S1**. The expression profiles of the various females, namely the list of mapped reads per each gene in each sample were clustered by two methods, hierarchical clustering (**Figure S2A**) and a principal component analysis (**Figure S2B**), which highlighted a large difference between D-cHH-injected animals and all the other groups, which in turn clustered together and did not display detectable differences in this preliminary analysis.

**Table 3:** Total number of sequencing reads obtained per animal and the number of the reads mapped and the corresponding percentage.

Sample	Total number of reads	Mapped reads (%)
N1	23,784,636	13,086,802 (55.0%)
N2	10,985,623	5,738,820 (52.2%)
N3	17,933,507	9,093,453 (50.7%)
S1	8,589,134	4,239,139 (49.4%)
S2	12,303,830	5,904,274 (48.0%)
S3	14,403,132	6,722,862 (46.7%)
L1	10,579,155	6,740,537 (63.7%)
L2	13,929,531	7,154,589 (51.4%)
L3	11,168,774	6,111,228 (54.7%)
D1	6,239,023	2,596,219 (41.6%)
D2	12,781,748	4,431,787 (34.7%)
D3	7,379,961	3,173,378 (43.0%)

N = native group, S = sham-injected, L = L-cHH injected and D = D-cHH injected.

**Table 4:** Number of differentially expressed transcripts in each of the experimental groups.

Group	Number of differentially expressed transcripts	Up regulated	Down regulated
S vs N	214	147	67
L vs S	45	30	15
D vs S	917	60	857

FDR corrected p-value <0.01, minimum difference in fold change = 2. S = sham-injected, L = L-cHH injected and D = D-cHH injected.

### Effects of XOSG removal on the hepatopancreas gene expression

The comparison between the S and N groups was useful to depict the effects derived solely from the XOSG removal procedure. XOSG extirpation resulted in the differential expression of 214 transcripts, with up-regulation (147 sequences, 68.7%) exceeding down-regulation (67 sequences, 31.3%). It is likely that the transcripts characterized by an increase of expression in respect to the naïve state are connected to two main events, the injury and the reduced production of several hormones caused by the XOSG removal. 63% of the up-regulated and 67% of the down-regulated genes showed no resemblance to known sequences. Among the genes which demonstrated similarity we identified in particular some transcripts connected to glycolysis process, such as transcript containing enolase domain (IPR000941), probably connected to the removal of cHHs production sites.

The hypergeometric test highlighted the carbohydrate metabolism (GO:0005975) and the chitinase activity (GO:0004568) as the most relevant GO terms repressed by XOSG extirpation; and many associated Interpro domains, e.g. glycoside hydrolase, family 9 (IPR001701), six-hairpin glycosidase (IPR012341) and chitinase II (IPR011583) were significantly repressed. Many other GO terms and Interpro signatures were found to be up-regulated, even though most of them could not be easily linked to any reported physiological effect of the extirpation. The most studied process in many crustacean species triggered by XOSG extirpation is undoubtedly vitellogenesis [61], [62], [63], but there is no uniform vitellogenesis-related physiological response to the extirpation among the studied species.

As for other genes, in pandalid shrimp, eyestalk ablation down-regulated the hepatopancreatic chitinase expression and also in *P. leptodactylus* we observed a significant repression of the IPR domain of the chitinase II (IPR011583) within the S group. This transcript pertains to the group 1 reported in Salma et al. [65] that represent chitinases produced in hepatopancreatic tissues that may function in the digestion of ingested chitin and the modification of peritrophic membrane in the intestine. Other studies [15], [64] showed that XOSG extirpation caused an increase in respiratory rate and a decrease in metabolic activity with respect to intact shrimps. Similarly, in *P. leptodactylus* the repression of six transcripts associated to metabolic process (GO:0008152) was observed in the S group: differently from *Marsupenaeus japonicus* [65] in which fructose-1,6-bisphosphatase was not significantly changed after XOSG removal, we observed an up-regulation of a transcript containing the fructose-1,6-bisphosphatase domain (IPR000146). This gene is involved in gluconeogenesis and cHH could affect also this metabolic pathway.

A similar although somewhat different experiment was carried out in the crayfish *Cherax quadricarinatus* by Yudkovski et al. [66]. Hepatopancreatic gene expression was compared between eyestalk extirpated and intact males. This experiment made use of a microarray analysis and differs from the present one by the time elapsed from extirpation to sacrifice (nine days vs two days here) and by the number of the reference genes which participated in the analysis (1865 vs 6,860 selected contigs here). Bearing in mind these differences, chitinases and carbohydrate metabolism-related enzymes were down-regulated in both studies. In addition, proteolytic enzymes and several hemocyanins were attenuated in the *C. quadricarinatus* experiment in contrary to the present experiment in which they were not differentially expressed. In general, the reduction in the hepatopancreas metabolic activity was more pronounced nine days after ablation than in P. leptodactylus two days after ablation.

XOSG removal affects also the circulating levels of hormones other than the cHH. These include the cHH type II hormones [7], the Molt Inhibiting Hormone (MIH), the Gonad/Vitellogenesis Inhibitng Hormone (GIH/VIH) and the Mandibular Organ-Inhibiting Hormone (MOIH). Hence, pathways controlled by these hormones may potentially be affected by the extirpation, an effect that would be difficult to discriminate due to the pleiotropic nature of the cHH members, affecting multiple processes [66].

### Differential effects of the two cHH-isomers on hepatopancreatic gene expression

The injection of the two cHHs, distinguished only by chirality, showed different effects on gene expression. The overall difference can be clearly demonstrated by the results of the clustering procedures revealing relatively different expression profile of the D-cHH-treated group in comparison to the other three groups, and in turn elucidated much more similar expression profiles with each other (Fig. S2). More in detail, comparison with the S group revealed only 45 differentially expressed transcripts in response to the administration of the L-isomer, whereas 917 transcripts were responsive to the D-isomer. Among the transcripts differentially up-regulated after L-cHH injection we depicted an increase of aminopeptidase n-like and trypsin 4, both correlated to proteolytic activity (GO:0006508), while all the down-regulated ones were without similarity. Moreover no functional or structural feature (GO term or domain) could be significantly linked to L-cHH injection by the hypergeometric test.

To examine a possible overlap in the effects exerted by L- and D-cHH we identified differentially expressed transcripts shared by the two treatments. Six transcripts elucidated differential expression caused by both treatments, but the majority of them had different trends of expression. No transcripts shared up-regulation caused by both treatments, while 2 non-annotated transcripts were mutually down-regulated (Pastle_hepa_c8804 and Pastle_hepa_c22565). Three transcripts were up-regulated after L-cHH, but down-regulated following D-cHH injection; among them a trypsin 4, a transcript containing chitinase II domain (IPR011583) and one without similarity. Only one non annotated transcript resulted to be up-regulated in D and down-regulated in the L group.

In the light of the results described above, the analysis was mainly focused on the variations between the S and the D groups.

**Table 5** presents the Interpro domains and the GO terms altered in the in D group in comparison to the S group as revealed by the hypergeometric test.

**Table 5:** List of the Interpro domains and GO terms differentially regulated by XOSG removal (S) or by D-cHH-injection (D), detected by the Hypergeometric test.

Group	Trend	Annotation	p-value	Description
S vs N	+	IPR006195	0.000117	Aminoacyl-tRNA synthetase, class II
S vs N	+	IPR002930	0.000979	Glycine cleavage H-protein
S vs N	+	IPR004365	0.000979	Nucleic acid binding, OB-fold, tRNA/helicase-type
S vs N	+	IPR020809	0.000979	Enolase, conserved site
S vs N	+	IPR020810	0.000979	Enolase, C-terminal
S vs N	+	IPR000537	0.00288	UbiA prenyltransferase family
S vs N	+	IPR000941	0.00288	Enolase
S vs N	+	IPR004364	0.00288	Aminoacyl-tRNA synthetase, class II (D/K/N)
S vs N	+	IPR003604	0.00288	Zinc finger, U1-type
S vs N	+	IPR018150	0.00288	Aminoacyl-tRNA synthetase, class II (D/K/N)-like
S vs N	+	IPR011053	0.00288	Single hybrid motif
S vs N	+	IPR001790	0.00564	Ribosomal protein L10/acidic P0
S vs N	+	IPR016027	0.00681	Nucleic acid-binding, OB-fold-like
S vs N	+	IPR012340	0.00681	Nucleic acid-binding, OB-fold
S vs N	+	IPR012675	0.00921	Beta-grasp domain
S vs N	+	GO:0005960	0.000979	Glycine cleavage compleX
S vs N	+	GO:0004659	0.000979	Prenyltransferase activity
S vs N	+	GO:0030414	0.00288	Peptidase inhibitor activity
S vs N	+	GO:0000287	0.00847	Magnesium ion binding
S vs N	+	GO:0004634	0.00921	Phosphopyruvate hydratase activity
S vs N	+	GO:0004867	0.00921	Serine-type endopeptidase inhibitor activity
S vs N	−	IPR001701	1.36E-05	Glycoside hydrolase, family 9
S vs N	−	IPR012341	1.89E-05	Six-hairpin glycosidase
S vs N	−	IPR008928	1.89E-05	Six-hairpin glycosidase-like
S vs N	−	IPR017853	0.000453	Glycoside hydrolase, superfamily
S vs N	−	IPR011583	0.0023	Chitinase II
S vs N	−	IPR001223	0.0023	Glycoside hydrolase, family 18, catalytic domain
S vs N	−	IPR013781	0.00314	Glycoside hydrolase, catalytic domain
S vs N	−	GO:0005975	0.000496	Carbohydrate metabolic process
S vs N	−	GO:0043169	0.000584	Cation binding
S vs N	−	GO:0003824	0.000858	Catalytic activity
S vs N	−	GO:0004568	0.0023	Chitinase activity
D vs S	+	IPR013128	0.0000548	Peptidase C1A, papain
D vs S	+	IPR013201	0.000777	Proteinase inhibitor I29, cathepsin propeptide
D vs S	+	IPR000668	0.0024	Peptidase C1A, papain C-terminal
D vs S	+	GO:0008234	0.000113	Cysteine-type peptidase activity
D vs S	+	GO:0004197	0.00544	Cysteine-type endopeptidase activity
D vs S	−	IPR000941	0.0058	Enolase
D vs S	−	IPR011042	0.0058	Six-bladed beta-propeller, TolB-like
D vs S	−	GO:0006096	0.000818	Glycolysis
D vs S	−	GO:0030234	0.00444	Enzyme regulator activity

+ designates up-regulation and − down-regulation.

As previously shown by the PCA analysis, the D-cHH administration caused pronounced alterations in comparison to the sham injected females, dominated by down-regulated genes (857 transcripts, accounting for 93.5% out of the total). The hypergeometric test elucidated a few D-cHH up-regulated functions, and in particular several GO terms related to peptidases, including cysteine-type peptidase (GO:0008234) and endopeptidase activity (GO:0004197), the papain domain of peptidase C1A, (IPR013128) and the cathepsin propeptide domain of proteinase inhibitor I29 (IPR013201), which is usually found in the N-terminus of many proteinases.

On the contrary, glycolysis (GO:0006096) appeared to be the GO term most significantly repressed in the D-cHH-injected female crayfish, which may be related to the return of the glycemic level to the native state, and suggests a modulation activity of the D-cHH on the glucose and energy metabolism in the hepatopancreas.

**Figure 2:**
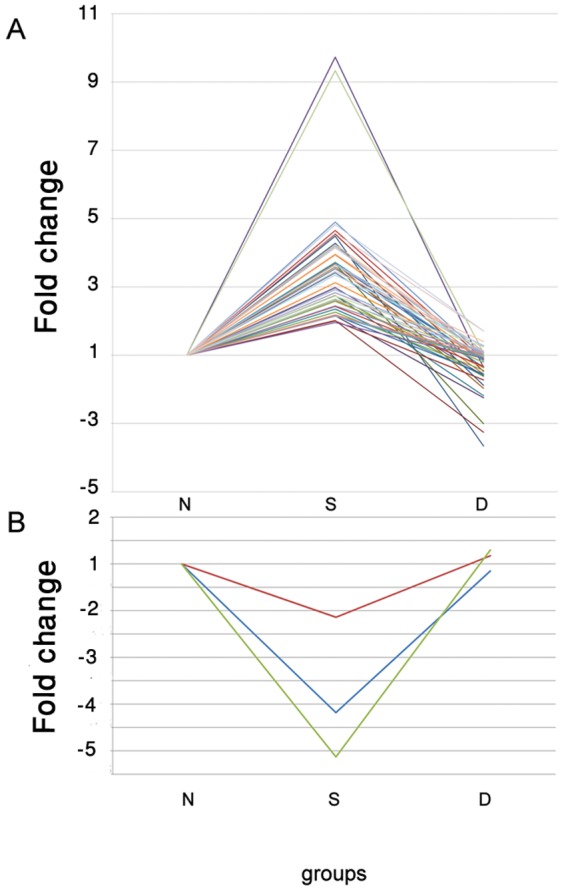
Expression trend of transcripts altered by XOSG ablation and restoring their native level following D-cHH injection. **A** Up-regulated transcripts following XOSG ablation. **B** Down-regulated transcripts following XOSG ablation.

Given the absence of the source of endogenous cHH, it was of interest to assess whether the injection of exogenous hormone was able to restore, even partially, the native conditions. Obviously, XOSG-ablation does not only remove the primary organ of cHH synthesis (cHH type I), but also prevents the secretion of other cHH superfamily hormones (cHH type II), possibly causing a series of gene expression changes not solely related to the deprivation of cHH itself. Therefore, the administration of synthetic cHH was only expected to partially restore the gene expression profile observed prior to XOSG ablation. In fact, about 1/3^th^ (50 out of 147) of the up-regulated transcripts in the S group were restored at lower level following the D-cHH injection (**Figure 2A**). Among them transcripts related to several different cellular processes and molecular functions were identified, including cellular organization (i. e. actin and tubulin beta 2), transcripts related to RNA processing and protein synthesis, (i.e, mRNA turnover protein 4-like protein), a transcript containing Fibrillarin domain (IPR000692) that plays a role in ribosomal RNA processing and lysyl-tRNA synthetase. Two cuticle proteins were also detected, playing a role in calcium ion binding; an armet-like protein precursor containing EF-Hand 1, calcium-binding site domain (IPR018247) and crustin 1 antimicrobial peptide were also restored by the D-cHH injection. Figure 2A shows two transcripts with an expression fold-change higher than the others, one encoding a C-reactive protein (CRP) containing a pentaxin domain (IPR001759) and the other transcript coding for a cuticle protein which contains the Rebers and Riddiford arthropod chitin binding domain (IPR000618). CPR is a fundamental player in the inflammatory response, in particular binding to the phosphocholine expressed on the surface of dead or dying cells in order to activate the complement system via the C1Q complex. Eyestalk ablation did not cause an increase in the level of CPR mRNA in *M. japonicus* in the ovary [67]. In the present study and in a different tissue an up-regulation of about 9.5-fold was depicted following the XOSG-ablation, subsequently restored by the D-cHH administration. But most notably, the expression of four transcripts crucially involved in glycolysis, which were increased in the S group, returned to lower levels after the D-cHH administration. Namely, phosphoglycerate mutase 1, two highly similar enolases catalyzing the eighth and ninth steps of glycolysis respectively, and fructose-1,6-bisphosphatase, an allosteric regulator of pyruvate kinase, the final step of glycolysis, all followed the same expression trends. This supports the hypothesis that D-cHH could have mainly an inhibitory effect on transcripts up-regulated following XOSG ablation and a minor effect on those silenced by the ablation, which probably required other molecules and centers of control located in the removed eyestalk. In fact, only 5% of the repressed genes upon ablation (3 out of 60) returned to the native levels of expression after D-cHH administration (**Figure 2B**). One of them resembles the 3-hydroxybutyrate dehydrogenase, related to oxydoreductase and metabolic processes and the two remaining transcripts have an unknown function.

The L-configuration provides the peptide with a strictly hyperglycemic activity whereas the D-cHH may exhibit other functions, in addition to a strong hyperglycemic potency. The finding of several transcripts containing a sodium symporter domain (IPR002657) suggests that D-cHH may also regulate glucose absorption by the hepatopancreas, a process that occurs through a Na^+^/D-glucose co-transport [68].

It has to be emphasized that the experiment was not intended to mimic natural situation in which the two hormonal species may be present in different levels, not necessarily reflected the injected amounts, but to apply rather pharmacological dose aimed at distinguishing the effects of the two isoforms. The data all together suggest that, despite being apparently able to restore glycemia to almost normal levels in eye-ablated animals one hour post injection (see **Table 1**), L-cHH does not significantly influence gene expression in the hepatopancreas. Therefore the apparent positive effect of the two neuropeptides on glycemia, at least after a short period, is either provided by a specific action of different tissues, or more likely still on the hepatopancreas, but through different mechanisms, one involving gene expression (D-cHH) and the other involving post translational enzyme activation (L-cHH). It is also possible that the effect of L-cHH on gene expression would be only visible at longer periods from its injection, after more than one hour. An additional experiment, similar to the one described here, but implementing a prolonged time-course and sampling of additional tissues has already been performed in our laboratory, and is currently being analyzed to provide better insight to the presented alternative hypotheses.

## Conclusions

The effects of two cHH enantiomers on a multi-gene expression profile of *P. leptodactylus* were examined here. The presence of both cHH isomers has been demonstrated only in Astacoidea [69]. The scarcity of *P. leptodactylus* sequenced transcripts required the construction of a comprehensive transcriptomic library of its hepatopancreas, comprising 39,935 contigs obtained through Illumina sequencing. This library was used as a reference gene assembly in a RNA-seq gene expression experiment. The short term effect of two enantiomers of the peptidic hyperglycemic hormone (D-cHH and L-cHH) of *P. leptodactylus* on hepatopancreatic gene expression was examined at two levels, the specific gene level and the functional group level.

The overall effect of the different treatments was demonstrated by clustering the 12 individual transcript profiles, which resulted with prominent difference of the transcription profiles of the D-cHH treated females in respect to all other samples.

This overall effect is detailed as follows: The XOSG synthesizes and secretes multiple peptidic hormones, including D- and L-cHH. The effect of its removal on gene expression profiles revealed the differential expression of 214 transcripts, 147 up-regulated and 67 down-regulated. The carbohydrate metabolic activity was repressed as expected from the elimination of the glycemia inducing hormone.

The L-cHH-injected individuals revealed an expression profile similar to sham injected individuals. In contrary, D-cHH caused a considerable short term change in the hepatopancreatic gene expression profile. 917 transcripts were responsive to this isomer: 857 out of them were down regulated and only 60 were up-regulated. The functions mainly affected were the up-regulation of the proteolytic activity and the down-regulation of glycolysis. L-cHH caused much less pronounced effects, with only 45 differentially expressed genes (30 up-regulated and 15 down-regulated), without the detectable alteration of any specific function.

The hypothesized glycemia-related mechanisms of L- and D- cHH may involve protein activation with no changes in gene expression for both isomers and an additional D-cHH mechanism which involves changes in gene expression, which may be related or not to the increase of glucose levels in the circulation.

The D-cHH effect on gene expression was also demonstrated by the hypergeometric test, which distinguished an opposite trend related to the glycolysis which was revealed in the XOSG ablated group (S). The later rend was characterized by the up regulation of the enolase (IPR000941) that resulted to be significantly repressed after the D-cHH administration. Moreover, this short-time study showed that D-cHH was apparently able to restore the expression of a certain number of transcripts to the level of the naïve state, and it was also able to affect molecular pathways not altered by the XOSG ablation.

## Supporting Information

Figure S1 The distribution of BLAST 13 Top Hit Species matching *P. leptodactylus* contigs.
(TIF)

Figure S2 Clustering the 12 mapping profiles of individual females using hierarchical and principal component analyses. **A**: Hierarchical clustering applying three clustering protocols, complete and single linkages and group average. (S) – sham injected, (N) – intact, (L) – L-cHH-injected, (D) – D-cHH-injected. **B**: principal component analysis. Green – native, Red – sham, Blue – L-cHH-injected and Yellow – D-cHH-injected.
(TIF)

Table S1 Please take a look at the excel document named as Table S1.
(XLSX)
