# Supplementary figures and images for: Application of D-Crustacean Hyperglycemic Hormone Induces Peptidases Transcription and Suppresses Glycolysis-Related Transcripts in the Hepatopancreas of the Crayfish Pontastacus leptodactylus — Results of a Transcriptomic Study
Source: PLoS One. 2013 Jun 19;8(6):e65176. doi: 10.1371/journal.pone.0065176 (PMC3686806; doi:10.1371/journal.pone.0065176)

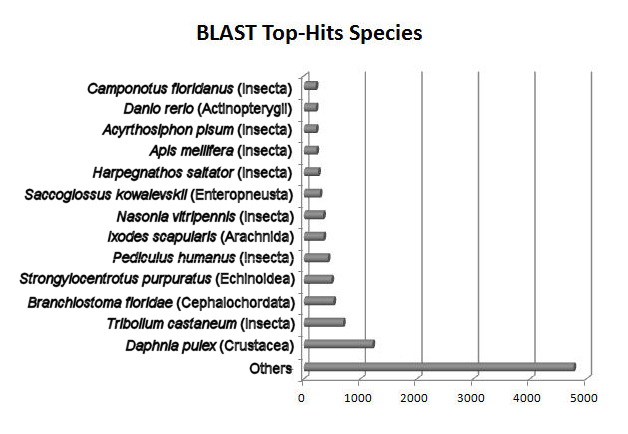

Supplement: Figure S1 — The distribution of BLAST 13 Top Hit Species matching P. leptodactylus contigs. (TIF) [file pone.0065176.s001.tif]

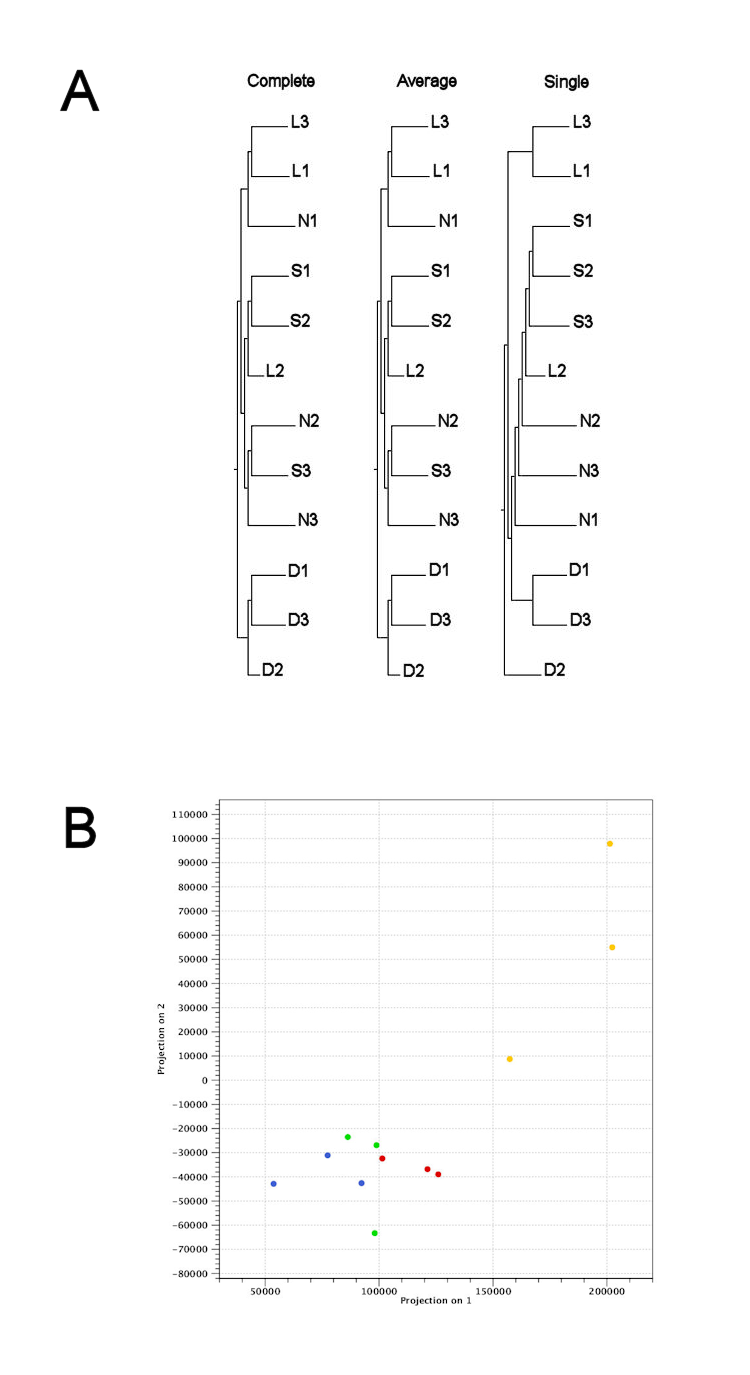

Supplement: Figure S2 — Clustering the 12 mapping profiles of individual females using hierarchical and principal component analyses. A: Hierarchical clustering applying three clustering protocols, complete and single linkages and group average. (S) – sham injected, (N) – intact, (L) – L-cHH-injected, (D) – D-cHH-injected. B: principal component analysis. Green – native, Red – sham, Blue – L-cHH-injected and Yellow – D-cHH-injected. (TIF) [file pone.0065176.s002.tif]
